# Estimating the prevalence and burden of major disorders of the brain in Nepal: methodology of a nationwide population-based study

**DOI:** 10.1186/1129-2377-15-52

**Published:** 2014-08-21

**Authors:** Kedar Manandhar, Ajay Risal, Timothy J Steiner, Are Holen, Rajendra Koju, Mattias Linde

**Affiliations:** 1 Department of Neuroscience, Norwegian University of Science and Technology, Trondheim, Norway; 2 Dhulikhel Hospital, Kathmandu University Hospital, Kavre, Dhulikhel, Nepal; 3 Division of Brain Sciences, Imperial College London, London, UK; 4 Pain unit, St Olavs University Hospital, Trondheim, Norway; 5 Norwegian National Headache Centre, St Olavs University Hospital, Trondheim, Norway

**Keywords:** Anxiety, Depression, Headache, Migraine, Prevalence, Burden of disease, Global campaign against Headache

## Abstract

**Background:**

The major disorders of the brain (MDBs), in terms of their prevalence and the burdens of ill health, disability and financial cost that they impose on individuals and society, are headache, depression and anxiety. No population-based studies have been conducted in Nepal.

**Aim:**

Our purpose was to assess the prevalence and burden attributable to MDBs in Nepal in order to inform health policy. Here we report the methodology.

**Methods:**

The unusual sociocultural diversity and extreme geographical variation of the country required adaptation of standard methodology. We ran pre-pilot and pilot studies before embarking on the main study. The study design was cross-sectional. The population of interest were adults aged 18–65 years who were Nepali speaking and living in Nepal. We selected, employed and trained groups of interviewers to visit randomly selected households by cold-calling. Households were selected from 15 representative districts out of 75 in the country through multistage cluster sampling. One participant was selected randomly from each household. We used structured questionnaires (the HARDSHIP questionnaire, Hospital Anxiety and Depression Scale, and Eysenck Personality Questionnaire -Neuroticism), culturally adapted and translated into Nepali. We recorded blood pressure, weight, height and waist circumference, and altitude of each household. We implemented various quality-assurances measures.

**Results:**

We completed the survey in one month, prior to onset of the monsoon. Among 2,210 selected households, all were contacted, 2,109 were eligible for the study and, from these, 2,100 adults participated. The participation rate was 99.6%.

**Conclusion:**

Standard methodology was successfully applied in Nepal, with some adaptations. The sociocultural and extraordinary geographic diversity were challenging, but did not require us to compromise the scientific quality of the study.

## Background

The major disorders of the brain (MDBs), in terms of their prevalence and the burdens of ill health, disability and financial cost that they impose on individuals and society, are headache, depression and anxiety [1-4]. Tension-type headache (TTH) and migraine are the second and third most prevalent disorders in the world [5], and migraine is the seventh highest specific cause of years of life lost to disability (YLDs) [5,6]. Major depressive and anxiety disorders are not as prevalent, but still common, and they contribute even more to disability at societal level: respectively they are the second and sixth leading specific causes of YLDs [5,7].

This is the global picture, but there are quite substantial regional uncertainties. Most epidemiological studies of the MDBs have been carried out in Western Europe and North America, so that their prevalence is poorly described, or unknown, in many large and populous regions elsewhere. This is nowhere more obvious than in the South-East Asia Region (SEAR), the only one of WHO’s six world regions for which no national data have been gathered so far of the societal impact of MDBs [8].

Nepal is a country of wide sociocultural diversity, while its three physiographic divisions attest its extraordinary geographical variation: from the Terai, or flat river plain of the Ganges in the south, at an altitude of below 300 m, through the central Hill division at 800–4,000 m, to the Himalayan Mountains above 4,000 m in the north containing eight of the world’s ten highest peaks [9]. In July 2011, the country’s population was estimated at almost 30 million [9], with nearly one quarter below the international poverty line of US$ 1.25 a day [9].

In such a country, how important are these MDBs? Very limited studies in Nepal have reported headache as one of the "major physical complaints" in both psychiatric and non-psychiatric clinics [10,11]. The Global Burden of Disease study 2010 (GBD2010) could only extrapolate to Nepal, since population-based data were not available from Nepal itself, but estimated that depressive disorders and migraine were each likely to be in the top five causes of disability, and anxiety in the top 20 [12]. This represents a lot of disability, in a country where life is a struggle. Yet, as elsewhere these conditions are neglected health problems [8].

The primary objective of this study was to estimate the prevalence and burden attributed to each of anxiety, depression and the headache disorders of public-health importance (migraine, TTH and medication-overuse headache [MOH]) in Nepal. The secondary objective was to identify the factors associated with each: demographic, life-style, comorbidities and health-care resource-utilization. The purpose was to support health policy through needs assessments, and provide evidence for health-care resource allocation in Nepal.

## Methods

### Ethics

The Nepal Health Research Council, the Institutional Review Committee of Kathmandu University School of Medical Sciences, Dhulikhel Hospital (IRC-KUSMS), and The Central Regional Committee for Health and Research Ethics in Norway all approved the study protocol.

Prospective participants who could do so read the written information approved by the ethics committees describing the nature and purpose of the study; to those who were illiterate, the interviewers read the information in the presence of family members. All had opportunity to ask questions. Before interviews commenced, consent was given in writing when possible but otherwise by fingerprint in accordance with requirements of IRC-KUSMS. All participants knew they were free to discontinue their interviews at any time.

Personal identification details of participants were separated from the completed questionnaires and stored in a locked room in the Department of Epidemiology at the Kathmandu University School of Medical Sciences. No information relating to identifiable individuals was disseminated beyond the researchers immediately involved in the study. All data were protected in accordance with Norwegian data-protection legislation.

### Study design

This was a cross-sectional, population-based survey using face-to-face structured interviews administered by trained interviewers. It was conducted by unannounced visits to households, with multistage random cluster sampling.

### Population of interest

The participants were adults aged 18–65 years who were Nepali speaking and living in Nepal. Excluded were immigrants (defined as those who had stayed for <6 months in the household and locality) and those who were deaf or otherwise unable to participate through major physical or mental health conditions.

### Sample size

We estimated a total sample size of 2,100, assuming a headache prevalence ≥10% and an absolute margin of error of 1.3% with 95% confidence interval [13].

### Sampling

To ensure an adequate representation from the country as a whole, we used a multistage random sampling technique described below. We divided the sample in the proportions 8:44:48 (numerically 170, 930, 1,000) between Mountain, Hill and Terai physiographic divisions according to their relative populations [14], and within each division equally between the five regions.

Furthermore, we aimed to recruit 25% of participants from above 2,000 m.

### Selection of districts

**Figure 1:**
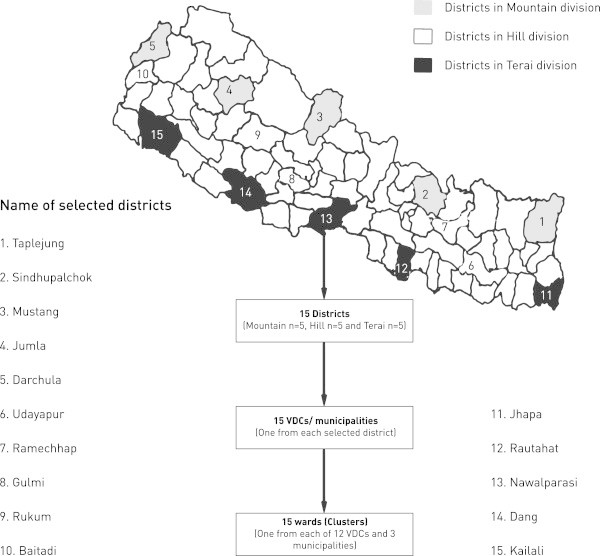
Flow chart of sites selection of the study.

The sampling procedure included each of the three physiographic divisions (Mountain, Hill and Terai) and five developmental regions (Far Western, Mid-Western, Western, Central and Eastern) dividing the country (Figure 1). By randomly selecting one district in each region of each division, we sampled 15 districts out the total of 75, spread across the country. We made one purposive change to the random selection, in the central developmental region of the Hill division, replacing Sidhuli by Ramechap because we needed one more cluster above 2,000 m.

### Selection of village development committees (VDCs) or municipalities

Each district consists of several village development committees (VDCs) in rural areas or municipalities in urban areas. We selected one of these local units from each district by a lottery sampling technique. Before doing this in the five districts in the Mountain division, VDCs or municipalities below 2,000 m were excluded so that sampling was done only from those above 2,000 m (representative of the mountainous terrain). In the Hill division, we applied an element of convenience sampling: two VDCs were selected purposively because access was very difficult to districts above 2,000 m in the selected districts (Ramechap and Rukum).

### Selection of clusters

Each VDC or municipality is built up of wards, these being collections of houses. In VDCs there are always nine wards, but in a municipality there may be more. We selected one ward per VDC or municipality, randomly by the lottery method, the households therein forming the clusters for final sampling (Figure 1).

### Selection of households

We defined a household as a group of people living together and sharing a common kitchen, and considered this as the sampling unit. A household in Nepal consists on average of 4.4 people [15]. A residential map of the cluster was drawn, excluding non-residential properties (empty or locked houses, store houses or animal sheds) with the help of the Female Community Health Volunteers (FCHVs) (local women working to improve community participation and enhance the outreach of health services at community level in Nepal). To select the first household we went to the center of the cluster and randomly selected a direction by the "spin the bottle" method. We then selected every household along the line in this direction until the projected sample size was achieved. If there were not enough households in the ward, the sampling continued in the same direction into the next adjoining ward of the same community.

### Selection of participants

In each household, the head, or a person who could provide reliable information, was asked to list all the family members within the age range 18–65 years. From this list, one was randomly invited to participate by the sealed envelopes method (this is a method of avoiding bias in selecting participants: the sealed envelope contains the numbers of family members, from which the head of the household picks one randomly). No replacements were permitted within a household for participants who withheld consent or failed to be available for interview.

### Recruitment and training of interviewers

We advertised in a national daily paper to find interviewers from throughout the country, selecting 47 of 150 applicants with backgrounds in health care, fluent in English and Nepali and with prior experience of population-based surveys. We divided them into eight teams according to their residence (knowledge of local language and customs) and appointed one member of each, with best skills, as team leader.

We provided a 4-day training programme at Dhulikhel Hospital on important aspects of the MDBs, on the principles of epidemiology and on the specific methods and requirements of the study. We observed them during simulated field interviews and gave feedback.

### Study instruments

For headache diagnosis and headache-attributed burden estimation, we used the Headache-Attributed Restriction, Disability, Social Handicap and Impaired Participation (HARDSHIP) questionnaire developed by LTB [6].This modular instrument included demographic enquiry and headache questions for all participants, then, for those reporting headache in the preceding year, diagnostic questions based on ICHD-3 beta followed by enquiries into the multiple components of burden [16]. We translated this into Nepali language following the LTB translation protocol for hybrid documents [17]. We administered the translated version in a pre-pilot study to 20 patients presenting at the Psychiatry Outpatient Department in Dhulikhel Hospital to verify cultural acceptability and inoffensiveness.

We imported Nepali-translated and culturally-validated versions of the Hospital Anxiety and Depression Scale (HADS) [18] and Eysenck Personality Questionnaire-Neuroticism (EPQ-N), revised version [19], into HARDSHIP (Additional file 1) to assess psychiatric morbidity in terms of caseness (a dichotomous measure) and symptom severity (a continuous measure).

We used a digital device (3BM1-3® by Microlife) to record blood pressure, with the participant sitting on the floor because many Nepalese would not have a chair in their homes. We used a simple portable scale to measure weight with shoes and outdoor clothes removed, and a measuring tape for height and waist circumference.

We used a portable altimeter (SAL 7030® by Sunoh) to establish the altitude of each household.

### Pilot study

The pilot study was a full test, in the field, of the survey methods and instruments. We convenience-selected Kavre district, with three clusters (Dhulikhel Municipality, Panchkhal and Kavre Bhanjyang), and set the sample size at 10% of that of the main study (*ie*, n = 210). Review during its progress and on completion led to modifications of the questionnaire and allowed assessment of the time required for data collection.

### Main survey

#### Engagement with participants

We ensured that each interviewer team included at least one member from the locality, and the assistance of FCHVs was sought in engaging with the community (Risal et al. submission of manuscript in Journal Headache and Pain).

Selected households were visited unannounced (cold-calling). Since most people were occupied with agriculture, most homes were locked during the day time. Interviewers therefore visited in the very early mornings and in the evenings. The survey was also continued during weekends (Saturday only in Nepal) and holidays. If the selected respondent was present and consented, the interview took place immediately. If he or she was not present at that time, a convenient appointment was made for a second visit, and, if necessary, a third. After three failed attempts, the selected person was registered as a non-participant.

### Quality assurance

We prepared a working manual for use by interviewer teams. In the field, completed questionnaires were reviewed by the team leader for completeness, accuracy and legibility at the end of each day. The team leader looked specifically for use of Arabic symbols for numbers (having different meanings in Nepali), mismatched age and gender of participants and differences between the list given by the head of household and the information from the participants. When minor mistakes were seen, the team leader corrected them after discussion with the interviewer; major mistakes or missing data missing were rectified by revisiting the household.

Every day while in the field, each team leader was required to contact the Nepali researchers (AR and KM) and report difficulties or queries. In addition, AR and KM made surprise visits to one of the two assigned sites of each team during data collection to ascertain that this was being done in accordance with expectations. We reviewed a random 10% sample of completed questionnaires and also cross-checked the collected data by revisiting 10% of households during the visit.

### Data management

All completed questionnaires were safely stored by the team leader in a plastic-coated box at the end of each day. After completion of data collection, they were sealed in double plastic bags and brought back to Dhulikhel hospital.

The data were entered by AR and KM into IBM SPSS Statistics version 20. Double entry of all data was made, with inconsistencies reconciled by reference to the original documents.

## Results

In the pre-pilot study (n = 20), the questionnaire was culturally acceptable and inoffensive. The pilot study led to modification of questions related to education, income, consumption and work, but identified no practical difficulties. Pilot study interviews did not contribute to the main analysis.

**Figure 2:**
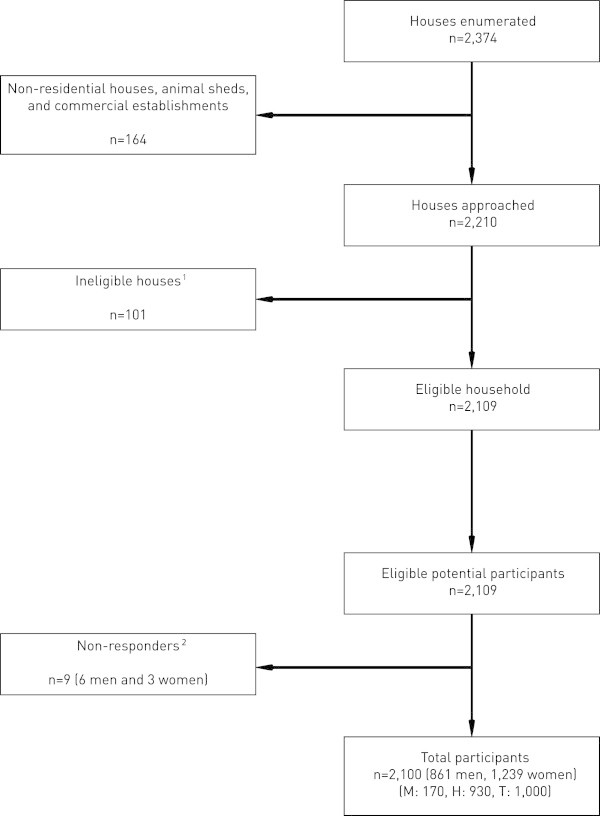
**Flow of participants.** (M = Mountain region, H= Hill region, T= Terai region). ^1^No one of age range of 18 to 65. ^2^Refused to consent or declined the interview.

The interviewers successfully reached every district and completed the main survey in one month (May 2013). The average time required per interview was approximately 45 minutes, and one hour per household to complete the data collection. There were no major communication problems. All 15 FCHVs were enthusiastically helpful in this regard.From 2,374 enumerated houses, 164 (non-residential, animal sheds and commercial establishments) were excluded (Figure 2). Among the remaining 2,210 households, 2,109 held eligible respondents, with one participant selected from each. There were nine (6 male, 3 female; age 20–40 years) who were classed as non-participants, four withholding consent altogether and five declining to complete the interview (these incomplete questionnaires were excluded from further analyses). Thus the participation rate was 99.6%.

Of the 2,100 participants, 1,239 (59%) were female and 861 (41%) were male (Table 1); their mean age was 36.4 ± 12.8 years. Males aged 18–34 years were under-represented in comparison with the national population of Nepal, according to census data (Table 1). Almost one quarter (491; 23.4%) of participants lived above 2,000 m.

**Table 1:** **Age and gender distributions in sample and population census 2011 of Nepal [**[19]**]**

**Age group (years)**	**Distribution of males in Nepali population (%)**	**Distribution of males in sample N (%)**	**Distribution of females in Nepali population (%)**	**Distribution of females in sample N (%)**
18-19	4.1^a^	61 (2.9)	4.2^a^	76 (3.6)
20-24	7.3	106 (5.0)	9.2	189 (9.0)
25-29	6.4	111 (5.3)	8.2	207 (9.9)
30-34	5.4	101 (4.8)	6.8	204 (9.7)
35-39	5.2	103 (4.9)	6.1	154 (7.3)
40-44	4.6	97 (4.6)	5.1	123 (5.9)
45-49	4.0	69 (3.3)	4.2	83 (4.0)
50-54	3.6	79 (3.8)	3.5	91 (4.3)
55-59	2.9	56 (2.7)	2.8	57 (2.7)
60-65	3.1^b^	78 (3.7)	3.3^b^	55 (2.6)
Total	46.6	861 (41.0)	53.4	1,239 (59.0)

^a:^Estimated as 0.4 x (population of age group 15–19 years).

^b:^Estimated as 1.2 x (population of age group 60–64 years).

Full results will be presented in future publications.

## Discussion

This study is the first nationwide population-based survey in Nepal to estimate the prevalence and burden of MDBs. The main purpose of this paper is to report how it was carried out in a country with rather unusual physiographic challenges, multiple languages and wide socioculture diversity. Because of the widespread illiteracy and lack of dependable communication services and poor infrastructure, a door-to-door survey with stratified, multistage cluster random sampling and face-to-face interviewing was the only feasible method. We carefully selected and trained interviewers for data collection. We used the well-established HARDSHIP questionnaire [6] with some cultural adaptations. It has a modular design and separate question sets to capture demographic characteristics, screen for caseness, diagnose headache type according to ICHD-3 beta criteria [16] and assess various quantifiable elements of burden. It has already been used successfully in population-based surveys of headache in China [20], India [21], Pakistan [22] and Russia [23] and proven to be valid and acceptable in similar sociocultural settings [21,22]. The Nepali-translated version appeared to be culturally acceptable and inoffensive. To measure anxiety and depression levels, HADS was incorporated into the HARDSHIP questionnaire as an additional module, both screening for caseness and assessing symptom severity. HADS is one of the practical and short rating scales that can capture both disorders, with good screening properties in the general population [24]. EPQ-N was also incorporated into the HARDSHIP questionnaire to explore the association between headache and neuroticism. It is a short and practical questionnaire with proven reliability and validity to measure the psychometric properties of neuroticism in an adult population in different socio-culture settings [25-27].

Nepal is home to many different ethnic groups; there are more than 100 indigenous languages [28,29]. The translation of all these instruments into multiple languages would be not only enormously resource-consuming but also of questionable value, because every translation would need validating and it was doubtful that the means to do this existed. Nepali is the *lingua franca* among the different ethno-linguistic groups [28,29], and the official language of Nepal, used in national surveys [15,30,14]. We believe the pragmatic decision to conduct the survey in Nepali was sensible, and vindicated by the participation rate of 99.6%.

This participation rate was exceptionally high, and a guarantee of freedom from participation bias [13]. Yet there was apparent under-representation of males aged 18–34 years, according to national census data from 2011. We believe this reflects different survey methods: we would have excluded family members temporarily resident outside the country as migrant workers (mostly young males), but the census might not [30,15]. Employing interviewers familiar with the local culture and sentiments, and who spoke the local language, and enlisting the help of FCHVs who were respected in the community, undoubtedly encouraged participation in the survey (Risal et al. submission of manuscript in Journal Headache and Pain), which exceeded that of studies recently conducted in other countries in Asia [20-22]. So, probably, did the strategy of data collection in early mornings, late evenings, weekends and public holidays. The very high participation rate in this study might also be attributed in part to the collaboration with Dhulikhel Hospital, well-known for its outreach health services throughout the country.

Quality assurance plays a vital role in estimating the prevalence of diseases and their burdens, especially when decisions are to follow on allocation of scarce health resources. Unfortunately, studies such as this are vulnerable to fraud, as has recently been shown [31]. Many quality-control measures were built into the methods of this study, beginning with careful selection and training of the interviewers. We prepared a working manual for the field. We planned and took preventive and detective measures throughout the study period: we were in daily mobile telephone communication with each team leader; we made surprise visits to sites during data collection; we checked data and households. This was labour-intensive work, adding cost to the study, but experience had shown it was important [31].

If our inability to validate the survey instruments was a limitation of the study, it was forced upon us. In the case of the headache diagnostic questions, there were no headache experts in Nepal to be the gold standard. This is a situation encountered elsewhere and, if it is seen as an insurmountable barrier, there is no way forward. While HARDSHIP reliably estimates the prevalence of *headache*, and the attribution of burden to headache disorders, which in public-health terms are of first importance, the relative contributions of migraine and TTH are also of considerable interest because somewhat different health-care provisions are needed for their management. The diagnostic questions of HARDSHIP are based on ICHD-3 beta [16], and have worked with good specificity and reasonable sensitivity (though less so for TTH) in several cultures and languages [6]. The diagnosis of MOH, or more correctly *probable* MOH, rests on the association in individuals of headache on ≥15 days/month and medication overuse, since there cannot be evidence of causation. The coexistence of these in itself signals ill health regardless of the precise diagnosis [32]. Similar considerations apply to HADS and EPQ-N. These are instruments that have been used and validated widely, and were used with the expectation, but not proof, that they would perform no less well in Nepal than elsewhere.

## Conclusion

Standard methodology [13] was applied in a nationwide population-based study estimating the prevalence and burden of major disorders of the brain in Nepal as a basis for needs assessment and to inform health policy. The sociocultural and extraordinary geographic diversity of this country presented challenges requiring some adaptations and a certain amount of ingenuity, but we did not need to compromise the scientific quality of the study.

## Abbreviations

DALY: Disability-adjusted life year; EPQ-N: Eysenck personality questionnaire-neuroticism; FCHV: Female community health volunteer; GBD2010: Global burden of disease survey 2010; HADS: Hospital anxiety and depression scale; HALT: Headache-attributed lost time; HARDSHIP: Headache-attributed restriction, disability, social handicap and impaired participation; IRC-KUSMS: Institutional review committee of Kathmandu University School of Medical Sciences, Dhulikhel Hospital; LTB: Lifting the burden; MDBs: Major disorders of the brain; MOH: Medication-overuse headache; SEAR: South-East Asia Region; TTH: Tension-type headache; VDC: Village development committee; WHO: World Health Organization; YLD: Year of life lost to disability.

## Competing interests

TJS is a Director and Trustee of *Lifting The Burden*.

## Authors' contributions

KM, AR, TJS, AH, RK, ML: Conception and design. Manandhar, Risal, Steiner, Linde: Acquisition of data. KM, AR, ML, TJS: Analysis and interpretation of data. Manandhar: Drafting the article. KM, AR, TJS, AH, RK, ML: Revising it critically for important intellectual content. AR, TJS, AH, RK, ML: Giving final approval of the version to be submitted. All authors read and approved the final manuscript.

## Supplementary Material

Additional file 1 HARDSHIP used in Nepal study. Click here for file
