# Supplementary material for: Estimating the prevalence and burden of major disorders of the brain in Nepal: methodology of a nationwide population-based study
Source: J Headache Pain. 2014 Aug 21;15(1):52. doi: 10.1186/1129-2377-15-52 (PMC4144696; doi:10.1186/1129-2377-15-52)
Supplement: Additional file 1 — HARDSHIP used in Nepal study. [file 1129-2377-15-52-S1.pdf]

Lifting The Burden

in Official Relations with
the World Health Organization

The Global Campaign against Headache

Headache-attributed restriction, disability, social handicap and impaired participation (HARDSHIP) questionnaire

Nepali version

for administration by trained nurses or health workers
to population samples

Respondent identifier
(to be completed by the interviewer)

☐	☐	☐☐	☐☐☐
enter letter to identify physiographic region<br>M: Mountain<br>H: Hill<br>T: Terai	enter letter to identify developmental area<br>F: Far west<br>M: Midwest<br>W: West<br>C: Central<br>E: East	enter 2-digit number to identify district	enter 3-digit number from master lists of households to identify household within sampling unit

from occupant list on next page:
enter 2-digit number 01-16 to identify household occupant ☐☐

Interviewer identifier
(to be completed by the interviewer)

____

Interviewer signature (on completion):

Respondent identification

Numbered list of household occupants

Enter given name, age and gender of each occupant in the order supplied

(age may be estimated if the date of birth is unknown)

	Given name	Age (y)	M/F
**01**			
**02**			
**03**			
**04**			
**05**			
**06**			
**07**			
**08**			
**09**			
**10**			
**11**			
**12**			
**13**			
**14**			
**15**			
**16**			

Open next envelope in numerical sequence from envelope-set for household size. This will contain a random number. Select the person with that number from the list above. The selected person will be the respondent.

Enter the number in the next column and on the previous page.

☐☐

enter 01-16 to identify selected household occupant

Thank you for answering the following questions. Please begin by entering **today's date**, and then **answer all questions on this day**.

1	Please enter today's date	______/______/_________

Demographic and social descriptor questions

2	What is your age?	______ **years**
3	What is your gender?<br>(please tick one box)	**male** ☐ **female** ☐
4	What is your native language (the language you first learned to speak)?	**enter name of language:**
5	What language do you usually speak in your own home?	**enter name of language:**
6	Which of these best describes your caste/ethnicity?<br>(please tick one box **only**)	☐ **Hill Brahman**<br>☐ **Hill Chhetri**<br>☐ **Tarai/Madhesi Brahman or Chhetri**<br>☐ **Tarai/Madhesi other castes**<br>☐ **Hill Dalit**<br>☐ **Tarai/Madhesi Dalit**<br>☐ **Newar**<br>☐ **Hill or Mountain Janajati**<br>☐ **Tarai Janajati**<br>☐ **Muslim**<br>☐ **Marwari, Bangali, Jain, Punjabi/Sikh**<br>☐ **Other**<br>☐ **Not applicable**
7	What is the altitude of the household?<br>(please enter reading from altimeter in meters)	___________ **meters**

**8**	What is the locality of the household?<br>(please tick one box **only**)	☐ **urban** ☐ **semi-rural** ☐ **rural**
**9**	What is your marital status?<br>(please tick one box **only**)	☐ **single** ☐ **married and living with spouse** ☐ **separated or divorced** ☐ **widowed**
**10**	What is the value of everything your **household consumes** in a year?<br>This may be the same as your household's total net income in money, or it may include the value of goods your household produces for itself, and/or goods and services paid for in kind (by exchange of other goods or services).<br>Please estimate the total as best you can, and tick one box.	**less than NPR 95,000** ☐<br>**between NPR 95,001 and NPR 120,000** ☐<br>**between NPR 120,001 and NPR 150,000** ☐<br>**between NPR 150,001 and NPR 240,000** ☐<br>**more than NPR 240,000** ☐
**11**	How much did your **household spend** during the **last month** on ordinary day-to-day living?<br>Do **not** include unusual expenses like family celebrations, annual holidays, purchase of large items (car, washing machine, etc).	**NPR** __________
**12**	Please consider everything your **household consumed** during the **last month** that you or your household produced for yourselves.<br>What was the value of these goods in money?	**NPR** __________
**13**	What is your level of literacy?<br>(please tick one box)	☐ **literate** ☐ **illiterate**
**14**	What is your level of your education?<br>(please enter grade, or tick one box)	☐ **no schooling** **school grade** ________ (enter grade 1-12) ☐ **graduate or higher**

**15**	Which of these is closest to your personal situation?<br>(please tick one box **only**)	**employed or self-employed** (go to question 16) ☐<br><br>☐ **household work** (enter code **996** at question 16)<br>☐ **student** (enter code **997** at question 16)<br>☐ **unemployed** (enter code **997** at question 16)<br>☐ **retired** (enter code **000** at question 16)
**16**	What is the nature of your work?<br>Please state the nature of your work.<br><br>Please refer to CBS Standard Occupational Classification. Enter the 3-digit code of the occupation that is closest to yours.	____________________<br>enter nature of work<br><br>☐☐☐<br>enter 3-digit CBS code

Thank you for answering these general questions. The remaining questions are about **your health**.

Screen questions

These next three questions are of crucial importance.

**17**	Have you **ever** had a headache **in your lifetime**?<br>(please tick one box)	**no** ☐ **yes** ☐<br>(if no, please still go to question 18)
**18**	Have you had a headache **during the last 12 months**?<br>(please tick one box)<br>**A negative answer terminates the enquiry into headache. Please ensure that the question is clearly understood.**	**no** ☐ **yes** ☐<br>(if no, go directly to question 90)
**19**	During **the last 30 days**, on how many of these days did you have a headache?<br>(please enter number of days between 0 and 30)	______ **days**<br>(if you answered between 15 and 30 days, please continue with question 20; otherwise, please go directly to question 27)

Daily headache questions

You have said that you had headache **on 15 or more days in the last month**. Please think about these headaches.

**20**	How long do these headaches usually last?<br>(please enter the number of minutes or hours, or tick the box)	____ **min or** ____ **hr** ☐ **never goes away**
**21**	Do you take **any medication** for these headaches?<br>(please tick one box)<br>(please note that this question is about treatment to **relieve** the headache, not daily treatment to prevent headache)	**no** ☐ **yes** ☐<br>(if no, go directly to question 24)
**22**	What medication **do you use most** for these headaches?<br>and what other medications do you also take for this purpose?<br>(if there are no others, please write "none")<br>(please note that this question is only about treatment to **relieve** headache)	**name the most-used medication:**<br>**list all other medications:**
**23**	Altogether, on how many days in the last 30 days did you take these medications?<br>(please enter number of days between 0 and 30)	______ **days**
**24**	Do you take **any herbal therapy** for these headaches?<br>(please tick one box)<br>(please note that this question is about herbal therapy to **relieve** the headache, not anything taken regularly to prevent headache)	**no** ☐ **yes** ☐<br>(if no, go directly to question 27)
**25**	What herbal therapy **do you use most** for these headaches?<br>and what other herbal therapies do you also take for this purpose?<br>(if there are no others, please write "none")<br>(please note that this question is only about therapies to **relieve** headache)	**name the most-used herbal therapy:**<br>**list all other herbal therapies:**
**26**	Altogether, on how many days in the last 30 days did you take herbal therapies?<br>(please enter number of days between 0 and 30)	______ **days**

"Most bothersome headache" questions

These are questions on the headaches that **interfere most with your life**.

These headaches may be the same as the headaches you have just described, or they may be different headaches if you have more than one type of headache.

27	Please think about your headaches. Do you think they **are all of one type**, or are they of **more than one type**?<br>(please tick one box)	**one** ☐ **more than one** ☐

If you answered one, the next questions are to diagnose this headache. Please start at question 28.

If you answered more than one, from now on please focus upon the headache type that on the whole bothers you most (*ie*, interferes most with your life).

The next series of questions are intended to diagnose this type of headache. Please start at question 28.

Diagnostic questions

28	How often do you have **this type of headache**?<br>(please tick box or enter the number of days per month or per year)	☐ **every day** ____ **days/month** ____ **days/year**
29	How long does **this type of headache** usually last?<br>(please enter the number of minutes, hours or days, or tick the box)<br>(if the headache goes away during sleep, count the time until you wake up without it)	___ **min,** ___ **hr or** ___ **days**<br>**never goes away** ☐
30	Is your last answer with or without medication?<br>(please tick one box)	**with** ☐ **without** ☐<br>(if you answered "without medication", please go to question 32)
31	How long would it last **if you did not take medication**?<br>(please enter the number of minutes, hours or days)	___ **min,** ___ **hr or** ___ **days**
32	How bad is **this type of headache** usually?<br>(please tick one box)	☐ **not bad** ☐ **quite bad** ☐ **very bad**

33	There are many ways of describing a headache, but most are either throbbing or pressing.<br>Thinking still of **this type of headache**, which better describes the pain?<br>(please tick one box)	☐ **throbbing or pulsating** (this means varying in time with the heart beat) ☐ **pressing, squeezing or tightening**
34	Is the pain of **this type of headache** usually on only one side of the head?<br>(please tick one box)	**no** ☐ **yes** ☐
35	Does exercise (like walking or climbing stairs) tend to make it worse?<br>(please tick one box)	**no** ☐ **yes** ☐
36	Thinking still of **this type of headache**, how does it affect your ability to do day-to-day activities?<br>(please tick one box)	☐ **can do everything as normal** ☐ **cannot do some things** ☐ **can do nothing**
37	With **this type of headache**, do you usually feel nauseated (as though you may vomit or throw up)?<br>(please tick one box)	**no** ☐ **yes** ☐
38	With **this type of headache**, do you usually actually vomit (throw up)?<br>(please tick one box)	**no** ☐ **yes** ☐
39	When you have **this type of headache**, does daylight or other lighting bother you? In other words, do you prefer to be in the dark?<br>(please tick one box)	**no** ☐ **yes** ☐<br>(this question refers to **ordinary** levels of light, not bright lighting)
40	When you have **this type of headache**, does noise bother you? In other words, do you prefer to be in the quiet?<br>(please tick one box)	**no** ☐ **yes** ☐<br>(this question refers to **ordinary** levels of noise, not very loud noise)

Thank you for answering these questions about your most bothersome headaches. The next series of questions are specifically about **yesterday** (the day before you fill in your answers).

It is very important that the answers you give are about **yesterday** and not any other day.

Questions about yesterday

41	Did you have a **headache yesterday**?<br>(please tick one box)	**no** ☐ **yes** ☐<br>(if no, go directly to question 53)
42	Was this the type of headache you have just been describing?<br>(please tick one box)	**no** ☐ **yes** ☐
43	Please think about the **headache you had yesterday**. How long did it last?<br>(please tick the box if it was present all day, from waking in the morning until bedtime, or enter the number of hours between 1 and 24)	**all day** ☐ **or** ______ **hours**
44	How bad was this **headache yesterday**?<br>(please tick one box)	☐ **not bad** ☐ **quite bad** ☐ **very bad**
45	Please think about everything you wanted to do **yesterday** if you had not had a headache.<br>How much of this did you **actually do**?<br>(please tick one box)	☐ **nothing** ☐ **less than half** ☐ **more than half** ☐ **everything**
46	Was **yesterday** a workday (either at your job or at school)?<br>(please tick one box)	**no** ☐ **yes** ☐<br>(if no, go directly to question 50)
47	Because of your headache, did you miss work or school **yesterday**?<br>(please tick one box or enter the number of hours lost from work or school)	☐ **no**<br>**arrived late, took time out during the day or left early** (please enter the total number of hours lost)**:** __________ **hours**<br>☐ **missed the whole day** (please go to question 50)
48	If you were at work or school with your **headache yesterday**, how much of your work did you get done?<br>(please tick one box))	☐ **nothing** ☐ **less than half** ☐ **more than half** ☐ **everything** (please go to question 50)

<table>
<tr><td>49</td><td>Will you able to make up for this today or later?
(please tick one box)</td><td colspan="4">☐ no   ☐ partly   ☐ completely</td></tr>
<tr><td>50</td><td>Please think about household work or general chores that you wanted to do yesterday if you had not had headache.
How much of this did you actually do?
(please tick one box)</td><td>☐
nothing</td><td>☐
less than half</td><td>☐
more than half</td><td>☐
everything</td></tr>
<tr><td>51</td><td>Please think about leisure and social activities that you wanted to do yesterday if you had not had headache.
How much of this did you actually do?
(please tick one box)</td><td>☐
nothing</td><td>☐
less than half</td><td>☐
more than half</td><td>☐
everything</td></tr>
<tr><td>52</td><td>What treatment did you take for the headache you had yesterday?
(please tick the box if you took nothing; otherwise, please list the names of all medications or herbal therapies taken for headache yesterday, and the number of times each was taken yesterday)
(please note that this question is only about treatment to relieve the headache, not daily treatment to prevent headache)</td><td colspan="4">nothing at all ☐

List medications:
(please list medications for headache, not for any other illnesses)
______________________ ______
______________________ ______
______________________ ______
______________________ ______
how many times you took each

List herbal therapies:
(please list therapies for headache, not for any other illnesses)
______________________ ______
______________________ ______
______________________ ______
how many times you took each</td></tr>
</table>

Thank you for answering these questions about headache yesterday. The next questions are about **all of your headaches**.

Health care questions

The aim of the following questions is to help us know how much health care should be available to meet the needs of people with headache.

53 Many people with headache treat themselves, but others need professional advice.

Have you had professional advice about your headaches **in the last year**?

Who from, and how many times?

Please tick all boxes that apply and, for each ticked box, enter the number of times in the last year.

		number of times
**no-one**	☐	
**health assistant, community medical assistant, health worker**	☐	____
**nurse**	☐	____
**physical therapist (physiotherapist, osteopath, chiropractor)**	☐	____
**primary-care doctor (GP)**	☐	____
**headache specialist**	☐	____
**ear, nose and throat doctor**	☐	____
**eye doctor**	☐	____
**psychologist**	☐	____
**psychiatrist**	☐	____
**hospital emergency room**	☐	____
**homoeopath**	☐	____
**Ayuverdic practitioner**	☐	____
**traditional healer, faith healer, Shaman**	☐	____
**other (please specify):** ____________	☐	____
**other (please specify):** ____________	☐	____

54	**Medications to treat headache**<br>These questions are about medications to **relieve** headache, not anything taken regularly to prevent headache.<br>Many different medications may be used successfully to treat headache.<br>Some are prescription-only, whilst others can be bought over the counter.<br>Please look at these lists. Which of these have you used **in the last month?**<br>Please tick the box if you took nothing at all in the whole of the last month; otherwise, enter by each medication the number of days on which you used it in the last month.	**nothing at all** ☐<br>**paracetamol**<br>**aspirin**<br>**other NSAIDs**<br>**combinations of NSAID+paracetamol**<br>**codeine or dihydrocodeine**<br>**combinations of codeine and other analgesic**<br>**other opioids**<br>**ergotamine tartrate**<br>**dihydroergotamine**<br>**triptan (suma- or other)**<br>**domperidone**<br>**metoclopramide**<br>**other anti-emetics**	**number of days**<br>_____ (one per medication)
54 (cont)	Are there any other medications you have used to treat your headache **in the last month**?<br>Please enter the name of each other medication and, by each, the number of days on which you used it in the last month.	**Name(s) of medication(s):**<br>(please list medications for **headache**, not for any other illnesses)<br>__________<br>__________<br>__________	**number of days**<br>_____<br>_____<br>_____
55	Are there any **herbal therapies** you have used to treat your headache **in the last month**?<br>Please enter the name of each herbal therapy and, by each, the number of days on which you used it in the last month.	**Name(s) of herbal therapy(s):**<br>(please list therapies for **headache**, not for any other illnesses)<br>__________<br>__________<br>__________<br>__________	**number of days**<br>_____<br>_____<br>_____<br>_____

**56**	Medications to **prevent** headaches are usually taken daily. Are you taking any of these now?<br><br>Please tick the box if you have taken nothing at all in the whole of the last month; otherwise, please enter the name(s) and, by each one, for how long **in weeks or months** you have been taking it<br><br>(please remember to specify weeks or months)	**nothing at all** ☐<br><br>**how long taken?**<br>**beta-blockers** ______<br>**valproate or divalproex** ______<br>**topiramate** ______<br>**amitriptyline** ______<br>**flunarizine** ______<br>**pizotifen** ______<br>**other (specify)**<br>____________________ ______<br>**other (specify)**<br>____________________ ______
**57**	Most people with headache do not require any investigations, but occasionally these tests are done.<br><br>Because of your headaches, have you had any of these tests **in the last year**?<br>(please tick <u>all</u> that apply)	**brain imaging (CT or MRI)** ☐<br>**EEG** ☐<br>**x-rays of the neck** ☐<br>**eye tests (for glasses)** ☐<br>**blood tests** ☐
**58**	Have you, **in the last year**, been admitted to hospital **because of your headaches**?<br>(please tick one box and, if yes, enter the total number of days in hospital)	**no** ☐ **yes** ☐<br><br>**total number of days** ______

Impact questions

The next questions are about the **effects your headaches have on your own life**.

**59**	Have your headaches interfered with your **education**?<br>(please tick all boxes that apply **because of your headaches**)	☐ **no**	☐ **yes, I did less well**	☐ **yes, I did not attempt something**	☐ **yes, I gave up early**

**60**	Have your headaches affected your ability to work over your lifetime?<br>(please tick all boxes that apply **because of your headaches**)<br>(if this question is not applicable to you, please tick no and go directly to question 63)	**no**	☐
		**yes, I have done less well**	☐
		**yes, I have attempted less**	☐
		**yes, I have taken an easier job**	☐
		**yes, I have taken long-term sick leave**	☐
		**yes, I have retired early**	☐
		**yes, I am on a disability pension**	☐

**61**	Have your headaches reduced your **earnings**?<br>(please tick one box)	**no** ☐	**yes** ☐	
**62**	Do you feel that your employer and work colleagues understand and accept your headaches?<br>(please tick one box)	☐ **no**	☐ **partly**	☐ **yes, fully**
**63**	Do you feel that your family and friends understand and accept your headaches?<br>(please tick one box)	☐ **no**	☐ **partly**	☐ **yes, fully**
**64**	Do you avoid telling people that you have headaches?<br>(please tick one box)	**no** ☐	**yes** ☐	

**65**	Taking into account everything you do to treat your headaches, how well do you think you control them?<br>(please tick one box)	☐ **not at all**	☐ **a little**	☐ **quite well**	☐ **completely**

The next questions are about **lost time because of your headaches**.		
**66**	On how many days **in the last 3 months** could you **not go to work or school** because of your headaches?<br>(please enter the number of days missed completely)	______
**67**	On how many days **in the last 3 months** could you do **less than half** your usual amount in your job or schoolwork because of your headaches?<br>(please enter the number of days; do **not** include days you counted in question 57 where you missed work or school)	______
**68**	On how many days **in the last 3 months** could you not do any household work because of your headaches?<br>(please enter the number of days lost completely)	______
**69**	On how many days **in the last 3 months** could you do **less than half** your usual amount of household work because of your headaches?<br>(please enter the number of days; do **not** include days you counted in question 59 where you did not do any household work)	______
**70**	On how many days **in the last 3 months** did you miss family, social or leisure activities because of your headaches?<br>(please enter the number of days)	______

The next questions aim to understand how much your headaches affect you **even when you do not actually have an attack**.

Please think carefully about the last day when you did **not** have a headache.

**71**	When was the last day when you did **not** have a headache?<br>(if you had no headache yesterday, please enter 1 day; otherwise enter the number of days or weeks since your last headache; if you cannot remember, tick the box and go directly to question 75))	______ **days** ______ **weeks** ☐ **cannot remember**
**72**	**On that day**, were you anxious or worried about your next headache?<br>(please tick one box)	**no** ☐ **yes** ☐
**73**	**On that day**, was there anything you could not do or did not do because you wanted to avoid getting a headache?<br>(please tick one box)	**no** ☐ **yes** ☐
**74**	**On that day**, did you feel **completely free** from all headache symptoms?<br>(please tick one box)	**no** ☐ **yes** ☐

The next questions ask about **willingness to pay for treatment**.

Imagine that there is a treatment you can buy. If you take it, your headaches will no longer bother you. How much would you be willing to pay **every month** for this treatment?

**75**	Would you pay NPR 100 a month?<br>(tick one box)<br>If the answer is no, go to question 76; if the answer is yes, go to question 79.	**no** ☐ **yes** ☐
**76**	Would you pay NPR 40 a month?<br>(tick one box)<br>If the answer is no, go to question 77; if the answer is yes, agree an amount between NPR 40 and NPR 100 and go directly to question 83.	**no** ☐ **yes** ☐<br>**agreed amount: NPR** ________
**77**	Would you pay NPR 20 a month?<br>(tick one box)<br>If the answer is no, go to question 70; if the answer is yes, agree an amount between NPR 20 and NPR 40 and go directly to question 83.	**no** ☐ **yes** ☐<br>**agreed amount: NPR** ________
**78**	Would you pay anything?<br>(tick one box)<br>If the answer is no, go directly to question 83; if the answer is yes, agree an amount between NPR 0 and NPR 20 and go directly to question 83.	**no** ☐ **yes** ☐<br>**agreed amount: NPR** ________
**79**	Would you pay NPR 200 a month?<br>(tick one box)<br>If the answer is yes, go to question 80; if the answer is no, agree an amount between NPR 100 and NPR 200 and go directly to question 83.	**no** ☐ **yes** ☐<br>**agreed amount: NPR** ________
**80**	Would you pay NPR 400 a month?<br>(tick one box)<br>If the answer is yes, go to question 81; if the answer is no, agree an amount between NPR 200 and NPR 400 and go directly to question 83.	**no** ☐ **yes** ☐<br>**agreed amount: NPR** ________
**81**	Would you pay NPR 1,000 a month?<br>(tick one box)<br>If the answer is yes, go to question 82; if the answer is no, agree an amount between NPR 400 and NPR 1,000 and go directly to question 83	**no** ☐ **yes** ☐<br>**agreed amount: NPR** ________
**82**	Would you pay NPR 2,000 a month?<br>(tick one box)<br>If the answer is yes, agree an amount of NPR 2,000 or more; if the answer is no, agree an amount between NPR 1,000 and NPR 2,000.	**no** ☐ **yes** ☐<br>**agreed amount: NPR** ________

The next three questions are about the effects your headaches have on **your relationships, love life and family planning**.

Please answer no to any that do not apply.

**83**	Have your headaches affected your family planning?<br>(please tick all boxes that apply **because of your headaches**)	**no** ☐<br>**yes, I have had fewer children** ☐<br>**yes, I have avoided having children** ☐<br>**yes, they have made it harder to conceive** ☐<br>**yes, I have avoided oral contraception** ☐
**84**	In **the last 3 months**, have your headaches caused difficulties in your love life?<br>(please tick one box)	**no** ☐ **yes** ☐
**85**	Have your headaches caused a relationship to break down?<br>(please tick one box)	☐ **no** ☐ **caused separation** ☐ **caused divorce**

The next two questions are for **people with children of school age**.

If they do not apply, please go directly to question 88.

**86**	During **the last 3 months**, have **your** headaches caused one or more of your children to miss school?<br>(please tick one box and, if yes, estimate the total number of missed days)	**no** ☐ **yes** ☐<br>**total number of days** ________
**87**	During **the last 3 months**, have **your** headaches prevented you from taking an interest in your children?<br>(please tick one box **only**)	☐ **less than once a month** ☐ **yes, once or more a month** ☐ **yes, once or more a week** ☐ **yes, every day**

The next two questions are for **people living with husband or wife**.

If you are not married, or not now living with your husband or wife, please go directly to question 90.

**88**	During **the last 3 months**, have **your** headaches caused **your husband or wife** to lose time from work?<br>(please tick one box and, if yes, enter the total number of days lost)	**no** ☐ **yes** ☐<br>**total number of days** _____
**89**	During **the last 3 months**, have **your** headaches caused **your husband or wife** to miss social activities?<br>(please tick one box and, if yes, enter the total number of occasions missed)	**no** ☐ **yes** ☐<br>**number of occasions** _____

The remaining questions are to be **answered by everyone**, with or without headaches.

General fitness questions

These measurements will give an indication of your level of fitness.

**90**	What is your weight?<br>(please enter weight **in ordinary clothing**, without shoes, in kilograms)	_________ **kg**
**91**	What is your height?<br>(please enter height, **without shoes**, in centimeters)	_________ **cm**
**92**	What is your waist measurement?<br>(please measure at the level of the umbilicus (navel) and enter the measurement in centimeters)<br>Tick the box if known to be pregnant.	________ **cm** ☐ **pregnant**
**93**	What is your blood pressure?<br>Please take one or two measurements following these instructions:<br>• from the right arm (unless this is not possible for any reason)<br>• with the arm elevated so that the cuff is at heart level<br>• with the subject sitting with feet on floor<br>• after 5 minutes' rest	______/______ **mm Hg**<br>Please repeat after 2 minutes **only** if the first measurement is above 140/90:<br>______/______ **mm Hg**

Quality of life questions (WHOQoL-8)

The questions ask how you feel about **your quality of life**, health or other areas of your life.

Each question has five response options. **Please choose the answer that appears most appropriate by circling the number in the appropriate column.** If you are unsure about which response to give to a question, the first response you think of is often the best one.

Please keep in mind your standards, hopes, pleasures and concerns. We ask that you think about your life **in the last 4 weeks.**

		very poor	poor	neither poor nor good	good	very good
**94**	How would you rate your quality of life?	1	2	3	4	5
		**very dissatisfied**	**dissatisfied**	**neither satisfied nor dissatisfied**	**satisfied**	**very satisfied**
**95**	How satisfied are you with your health?	1	2	3	4	5
**96**	How satisfied are you with your ability to perform your daily living activities?	1	2	3	4	5
**97**	How satisfied are you with yourself?	1	2	3	4	5
**98**	How satisfied are you with your personal relationships?	1	2	3	4	5
**99**	How satisfied are you with the conditions of your living place?	1	2	3	4	5
		**not at all**	**a little**	**moderately**	**mostly**	**completely**
**100**	Do you have enough energy for everyday life?	1	2	3	4	5
**101**	Have you enough money to meet your needs?	1	2	3	4	5

Depression and anxiety questions (HADS)

These 14 questions ask about **depression and anxiety**, both of which are common in the general population.

Each question has four response options. **Please choose the answer that appears most appropriate by circling the number in the appropriate column.** If you are unsure about which response to give to a question, the first response you think of is often the best one.

		**Most of the time**	**A lot of the time**	**Time to time, occasionally**	**Not at all**
**102**	I feel tense or "wound up"	3	2	1	0
		**Nearly all of the time**	**Very often**	**Sometimes**	**Not at all**
**103**	I feel as if I am slowed down	3	2	1	0
		**Definitely as much**	**Not quite so much**	**Only a little**	**Not at all**
**104**	I still enjoy the things I used to enjoy	0	1	2	3
		**Not at all**	**Occasionall y**	**Quite often**	**Very often**
**105**	I get a sort of frightened feeling like "butterflies in the stomach"	0	1	2	3
		**Very definitely and quite badly**	**Yes, but not too badly**	**A little, but it doesn't worry me**	**Not at all**
**106**	I get a sort of frightened feeling like something awful is about to happen	3	2	1	0
		**Definitely**	**I don't take as much care as I should**	**I may not take quite as much care**	**I take just as much care as ever**
**107**	I have lost interest in my appearance	3	2	1	0

		As much as I always could	Not quite so much now	Definitely not so much now	Not al all
108	I can laugh and see the funny side of things	0	1	2	3
		**Very much indeed**	**Quite a lot**	**Not very much**	**Not at all**
109	I feel restless as if I have to be on the move	3	2	1	0
		**A great deal of the time**	**A lot of the time**	**From time to time but not too often**	**Only occasionally**
110	Worrying thoughts go through my mind	3	2	1	0
		**A much as I ever did**	**Rather less than I used to**	**Definitely less than I used to**	**Hardly at all**
111	I look forward with enjoyment to things	0	1	2	3
		**Not at all**	**Not often**	**Sometimes**	**Most of the time**
112	I feel cheerful	3	2	1	0
		**Very often indeed**	**Quite often**	**Not very often**	**Not at all**
113	I get sudden feelings of panic	3	2	1	0
		**Definitely**	**Usually**	**Not often**	**Not at all**
114	I can sit at ease and feel relaxed	0	1	2	3
		**Often**	**Sometimes**	**Not often**	**Very seldom**
115	I can enjoy a good book or radio or TV programme	0	1	2	3

Personality questions (Eysenck)

The next 12 questions ask about **the sort of person you are**.

Please answer each question "yes" or "no". Answer quickly; do not spend a long time thinking about the exact meaning of the questions.

**116**	Does your mood often go up and down?	**no** ☐	**yes** ☐
**117**	Do you ever feel "just miserable" for no reason?	**no** ☐	**yes** ☐
**118**	Are you an irritable person?	**no** ☐	**yes** ☐
**119**	Are your feelings easily hurt?	**no** ☐	**yes** ☐
**120**	Do you often feel "fed-up"?	**no** ☐	**yes** ☐
**121**	Would you call yourself a nervous person?	**no** ☐	**yes** ☐
**122**	Are you a worrier?	**no** ☐	**yes** ☐
**123**	Would you call yourself tense or "highly-strung"?	**no** ☐	**yes** ☐
**124**	Do you worry too long after an embarrassing experience?	**no** ☐	**yes** ☐
**125**	Do you suffer from "nerves"?	**no** ☐	**yes** ☐
**126**	Do you often feel lonely?	**no** ☐	**yes** ☐

**127**	Are you often troubled about feelings of guilt?	**no** ☐ **yes** ☐

Substance-use questions

These next five questions ask about **smoking, alcohol and marijuana** use.

**128**	Do you smoke **tobacco**? (please tick one or two boxes as applicable)	☐ **no** (go to question 131)	☐ **cigarettes** (go to question 129)	☐ **water pipe** (go to question 129)
**129**	How many cigarettes do you smoke each day on average? (please enter number)	**number per day** ________		
**130**	How many cigarettes did you smoke yesterday? (please enter number)	**number smoked yesterday** ________		
**131**	How often do you have a drink containing alcohol? (please tick one box **only**)	☐ **never**	☐ **rarely (less than once a month)**	☐ **2-5 times a month**
		☐ **2-3 times a week**	☐ **4-6 times a week**	☐ **every day**
**132**	Do you use **marijuana (hashish or cannabis)?** If so, how often on average? (please tick one box **only**)	☐ **never**	☐ **rarely (less than once a month)**	☐ **2-5 times a month**
		☐ **2-3 times a week**	☐ **4-6 times a week**	☐ **every day**

Load-carrying questions

Next are three questions about **use of a tumpline** (a sling formed by a strap over the forehead, commonly used in Nepal for carrying a pack on the back or in hauling loads).

**133**	Do you ever use a tumpline?<br>(please tick one box)	**no** ☐ **yes** ☐<br>(if no, go directly to question 136)				
**134**	How often, on average?<br>(please tick one box **only**)<br>This question relates to a **typical week**	☐ **less than once a week**	☐ **1 day a week**	☐ **2-3 days per week**	☐ **4-6 days per week**	☐ **every day**
**135**	How heavy are the loads carried or pulled?<br>(please tick one box **only**)<br>This question relates to a **typical week**	☐ **very light**	☐ **light**	☐ **moderate**	☐ **heavy**	☐ **very heavy**

And one final question about your life overall ...

**136**	How would you rate your life overall?<br>(please tick one box **only**)	☐ **very easy**	☐ **easy**	☐ **medium**	☐ **tough**	☐ **very tough**

The questionnaire is now complete. Thank you very much for your time.

**This section is only for respondents in the validation sub-sample.**		
137	Physician-diagnosis of most bothersome headache (if made)	______________________
138	Physician-diagnosis of other headache 1 (if made)	______________________
139	Physician-diagnosis of other headache 2 (if made)	______________________
